# Efficacy, treatment retention, and tolerability of newer- versus older-generation antiseizure medications in older adults with epilepsy: a systematic review and meta-analysis

**DOI:** 10.3389/fneur.2026.1902489

**Published:** 2026-09-21

**Authors:** Changyan Fan, Chaosheng Li, Chenyan Sui, Likun Han, Xin Gu, Yong Liu

**Affiliations:** 1 Department of Neurology, Affiliated Hospital of Jiangnan University, Wuxi, Jiangsu, China; 2 Department of Cardiology, Affiliated Hospital of Jiangnan University, Wuxi, Jiangsu, China

**Keywords:** antiseizure medications, epilepsy, meta-analysis, older adults, seizure freedom, treatment retention

## Abstract

**Objective:**

Older adults with epilepsy frequently have multiple comorbidities, polypharmacy, and reduced medication tolerance; therefore, ASM selection must consider seizure control, treatment persistence, and tolerability. This study compared the efficacy and safety outcomes of newer-generation and older-generation ASMs in older adults with epilepsy.

**Methods:**

PubMed, Embase, Web of Science Core Collection, and the Cochrane Library were searched from inception to April 30, 2026. Comparative clinical studies evaluating newer-generation versus older-generation ASMs in populations defined as older adults by the original studies were eligible. The primary outcome was seizure freedom; secondary outcomes included seizure-free retention, treatment retention, withdrawal for any reason, discontinuation due to adverse events, and adverse reactions. Risk ratios (RRs) with 95% confidence intervals (CIs) were pooled using fixed- or random-effects models according to statistical and clinical heterogeneity.

**Results:**

Nine studies involving 2,507 participants were included, comprising randomized, open-label, *post-hoc* subgroup, and retrospective evidence. Overall seizure freedom did not differ significantly between the two medication categories (RR = 1.18, 95% CI 0.80–1.76; *I*^2^ = 93%), and the restricted sensitivity analysis remained non-significant (RR = 1.11, 95% CI 0.94–1.30). No significant difference was observed in seizure-free retention. Newer-generation ASMs were associated with higher treatment retention (RR = 1.34, 95% CI 1.22–1.47), with the inverse outcome of withdrawal for any reason correspondingly lower (RR = 0.71, 95% CI 0.64–0.80); adverse-event-related discontinuation was also lower (RR = 0.54, 95% CI 0.45–0.64). In the exploratory post-stroke analysis based on two randomized studies, the random-effects estimate did not show a significant difference in seizure freedom (RR = 1.30, 95% CI 0.81–2.08; *I*^2^ = 77%).

**Conclusion:**

Across a small and clinically heterogeneous evidence base, seizure freedom did not clearly differ between newer- and older-generation ASMs, whereas treatment retention and adverse-event-related discontinuation favored newer-generation agents; however, the certainty of evidence was low or very low. These class-level findings should be interpreted cautiously because the medication categories were pharmacologically heterogeneous and the evidence included mixed study designs, variable age definitions, and inconsistent outcome windows.

## 1 Introduction

Epilepsy is one of the more common chronic neurological disorders in older adults, and its disease burden continues to increase as population aging accelerates (1). Current epidemiological evidence indicates that epilepsy is a common chronic neurological disease worldwide, with an active epilepsy point prevalence of approximately 6.38‰ and a lifetime prevalence of approximately 7.60‰, suggesting that its disease burden remains substantial (2). Compared with epilepsy in children, adolescents, and young adults, epilepsy in older adults has a more complex etiological profile and more varied clinical manifestations. Stroke, neurodegenerative disease, traumatic brain injury, brain tumors, and metabolic abnormalities are all important factors associated with epilepsy in later life (3). Because seizure manifestations in older patients are often atypical, some cases may present as transient confusion, behavioral abnormalities, cognitive fluctuation, or nonspecific psychiatric symptoms. In clinical practice, these manifestations may be confused with syncope, transient ischemic attack, cognitive impairment, or psychiatric and behavioral disorders, increasing the risk of missed diagnosis, misdiagnosis, and delayed treatment (4). Epidemiological studies have also shown that the risks of acute symptomatic seizures and epilepsy in people over 60 years of age increase with advancing age, suggesting that epilepsy in older adults has become an important issue in chronic neurological disease management (5).

Drug therapy remains the mainstay of long-term management for epilepsy in older adults. Older-generation ASMs, such as carbamazepine, phenytoin, sodium valproate, and phenobarbital, have been used for many years. Because of their accumulated clinical experience and relatively low treatment costs, they continue to occupy an important place in epilepsy treatment (6, 7). However, with the progress of research, increasing evidence has shown that older patients often have reduced hepatic and renal function, altered plasma protein binding, multiple chronic diseases, and polypharmacy. Their pharmacokinetic characteristics and treatment tolerance therefore differ from those of younger patients. Some older-generation ASMs have enzyme-inducing properties or narrow therapeutic windows, which may increase the risks of drug–drug interactions, cognitive impairment, hyponatremia, abnormal bone metabolism, gait instability, and falls, ultimately affecting treatment adherence and long-term outcomes (8–10). In recent years, newer-generation ASMs such as lamotrigine, levetiracetam, gabapentin, and oxcarbazepine have increasingly been used in older adults with epilepsy. Although these medications are commonly grouped according to the period of their introduction, they differ in mechanism of action, elimination pathway, interaction potential, and adverse-effect profile; therefore, the category should not be regarded as pharmacologically homogeneous (11, 12). Even so, whether newer-generation drugs offer clear advantages over older-generation drugs in efficacy and safety still requires further systematic evaluation. In reviewing the literature, we found that previous studies have mainly focused on direct comparisons between individual drugs (13), or have analyzed older patients only as a subgroup within adult epilepsy studies (14). Such evidence is not always sufficient to guide treatment decisions specifically for older patients with epilepsy. For this population, whether a drug can be maintained over the long term, whether treatment is discontinued because of adverse reactions, and how well the drug is tolerated during therapy all have a direct impact on clinical benefit and deserve careful attention.

Against this background, the present meta-analysis compared seizure outcomes, treatment retention, and treatment discontinuation between newer-generation and older-generation ASMs in older adults with epilepsy. These terms were used as operational categories reflecting the comparisons reported in the eligible studies; the pooled estimates therefore describe an average class-level pattern and should not be interpreted as a ranking of individual ASMs.

## 2 Materials and methods

### 2.1 Literature search

PubMed, Embase, Web of Science Core Collection, and the Cochrane Library were searched from database inception to April 30, 2026. The search combined controlled vocabulary and free-text terms relating to epilepsy, older adults, and ASMs. The medication component included class-level terms and individual medication names, including lamotrigine, levetiracetam, gabapentin, oxcarbazepine, topiramate, zonisamide, lacosamide, eslicarbazepine acetate, brivaracetam, carbamazepine, phenytoin, valproate, and phenobarbital. No language restriction was applied in the updated search. Reference lists of the included studies and relevant reviews were also screened. The complete database-specific strategies, including subject headings, field tags, truncation, and database-specific syntax, are provided in Supplementary Table S1. The review was not prospectively registered, and no publicly accessible protocol was available. This limitation is acknowledged because the subgroup and influence analyses could not be verified against a prospectively published protocol. The manuscript was prepared in accordance with the PRISMA 2020 and PRISMA-S reporting recommendations.

### 2.2 Inclusion criteria

Eligibility was defined according to the PICOS framework. The population comprised adults classified as older by the original study, including cohorts using minimum ages of ≥55, ≥60, or ≥65 years, as well as post-stroke cohorts whose participants were predominantly older adults. Studies enrolling broader adult populations were eligible only when the relevant comparative data represented an older or predominantly older cohort. The intervention was a newer-generation ASM, and the comparator was an older-generation ASM, as operationally classified in the eligible studies. The primary outcome was seizure freedom; secondary outcomes were seizure-free retention, treatment retention, withdrawal for any reason, discontinuation due to adverse events, and adverse reactions. Eligible designs included randomized controlled trials, open-label randomized trials, *post-hoc* older-adult subgroup analyses, and retrospective comparative studies. The disease types included newly diagnosed epilepsy in older adults, new-onset epilepsy, focal epilepsy, post-stroke epilepsy, or other clearly diagnosed epilepsy-related populations in older adults; the newer-generation group used newer-generation ASMs, including lamotrigine, levetiracetam, gabapentin, oxcarbazepine, topiramate, zonisamide, and others; the older-generation group used older-generation ASMs, including carbamazepine, phenytoin, sodium valproate, phenobarbital, and others. Studies were required to report at least one extractable efficacy or safety outcome, such as seizure freedom rate, seizure-free retention rate, treatment retention rate, withdrawal rate for any reason, discontinuation rate due to adverse events, or incidence of adverse reactions. Age threshold, epilepsy etiology, seizure type, study design, follow-up duration, and treatment context were extracted for every study and were considered when interpreting clinical heterogeneity.

### 2.3 Exclusion criteria

Duplicate publications, overlapping data sources, or multiple reports based on the same study population were excluded. When several reports involved the same population, the study with the most complete data and the outcomes most relevant to this analysis was retained. Reviews, systematic reviews, meta-analyses, guidelines, expert consensus statements, case reports, animal experiments, and basic research that did not provide original clinically comparable data suitable for pooling were excluded. Studies with unclear outcome definitions, missing key data, or data that could not be obtained from the original text or by calculation were also excluded. Studies were further excluded if the full text was unavailable, or if the study design, grouping information, or outcome data were insufficient to determine eligibility.

### 2.4 Literature screening

The retrieved records were imported into Endnote X9 for deduplication. Two researchers independently screened titles and abstracts to exclude studies clearly inconsistent with the inclusion criteria; full texts were then reviewed and rescreened according to the inclusion and exclusion criteria. Any disagreement during screening was resolved through discussion between the two researchers. If consensus could not be reached, a third researcher was consulted.

### 2.5 Data extraction

Data were independently extracted by two researchers, and the extracted information was cross-checked. The extracted items included first author, publication year, study design, source of study population, age criteria, epilepsy type or etiology, sample size, drugs used in the newer-generation group, drugs used in the older-generation group, follow-up duration, efficacy outcomes, safety outcomes, and reasons for withdrawal or discontinuation. For multi-arm studies, the eligible newer-generation group and older-generation group were combined according to the grouping principle of this study, so that the same older-generation group would not be counted repeatedly. If a study reported multiple follow-up time points, the primary follow-up endpoint prespecified in the original study was preferentially extracted. If the original study did not specify a primary endpoint, the time point with clearer clinical significance and better comparability across studies was selected. For each study and outcome, the medication arms, event counts, denominators, follow-up period, operational definition, and inclusion in or exclusion from each meta-analysis were recorded. These data are presented in Supplementary Table S2.

### 2.6 Outcome indicators

The primary outcome was seizure freedom, defined according to the operational definition and assessment period used in each original study. Secondary outcomes were seizure-free retention, treatment retention, withdrawal for any reason, discontinuation due to adverse events, and adverse reactions. The exact definition, follow-up period, event count, denominator, and contribution of each study to each synthesis are presented in Supplementary Table S2. Outcomes were pooled only when they represented the same broad clinical construct; differences in follow-up duration and operational definition were treated as sources of clinical heterogeneity. Treatment retention and withdrawal for any reason were recognized as inverse and overlapping outcomes and were not interpreted as independent confirmation of the same association. Discontinuation due to adverse events was retained as a separate cause-specific outcome. Seizure-free retention rate was used to reflect seizure freedom while patients continued treatment; treatment retention rate was used to evaluate treatment persistence and clinical acceptability; withdrawal rate for any reason and discontinuation rate due to adverse events were used to reflect overall withdrawal risk and safety-related discontinuation during treatment. For patients with post-stroke epilepsy, subgroup analysis was further performed to compare the efficacy and safety of newer-generation and older-generation ASMs in this specific etiological population.

### 2.7 Quality assessment

Quality assessment tools were selected according to study design. Randomized controlled trials were evaluated using the Cochrane risk-of-bias tool, covering random sequence generation, allocation concealment, blinding of participants and researchers, blinding of outcome assessment, completeness of outcome data, selective reporting, and other biases. Non-randomized studies, retrospective cohort studies, and real-world studies were assessed using the Newcastle-Ottawa Scale (NOS), mainly from the perspectives of study population selection, comparability between groups, and outcome assessment. Quality assessment was independently performed by two researchers. Disagreements were resolved through discussion, or, when necessary, by a third researcher. Study-level judgments for every risk-of-bias domain and complete NOS assessments are presented in Supplementary Table S3. When methodological information was not reported sufficiently in the original article, the corresponding domain was rated as unclear rather than inferred to be at low risk. The certainty of evidence for each outcome was assessed using the GRADE framework, considering risk of bias, inconsistency, indirectness, imprecision, and publication bias. The complete evidence profile is presented in Supplementary Table S4.

### 2.8 Statistical analysis

RevMan 5.4 was used for meta-analysis. All outcome indicators were dichotomous variables, and effect sizes were expressed as risk ratios (RRs) with 95% confidence intervals (CIs), pooled using the Mantel–Haenszel method. For positive outcomes such as seizure freedom rate, seizure-free retention rate, and treatment retention rate, RR > 1 indicated a higher event rate in the newer-generation group; for negative outcomes such as withdrawal rate for any reason, discontinuation rate due to adverse events, and incidence of adverse reactions, RR < 1 indicated a lower related risk in the newer-generation group. *p* < 0.05 was considered statistically significant. Heterogeneity among studies was evaluated using the *χ*^2^ test and *I*^2^ statistic. Model selection considered both statistical and clinical heterogeneity. Random-effects models were used when substantial between-study heterogeneity or clearly different clinical contexts were present. Fixed-effect models were retained for treatment retention, withdrawal for any reason, and adverse-event-related discontinuation because these analyses used closely aligned event definitions and showed low-to-moderate statistical heterogeneity; random-effects sensitivity analyses were additionally performed for these outcomes. The post-stroke outcomes were analyzed using random-effects models because the studies differed in design and showed substantial heterogeneity. Because randomized and non-randomized evidence are subject to different sources of bias, sensitivity analyses excluding retrospective studies were performed for outcomes to which retrospective studies contributed. Separate pooling of observational studies was not undertaken when only one observational study contributed to an outcome.

### 2.9 Sensitivity analysis and subgroup analysis

Sensitivity analysis was performed for the primary outcome and outcomes with heterogeneity. Sensitivity was examined by removing each study in turn and recalculating the pooled RR and *I*^2^. Additional exploratory restricted analyses were conducted when individual studies showed markedly divergent effect estimates or differed materially in design or outcome definition. All exclusions, rationales, and resulting estimates are reported in Supplementary Table S5; these analyses were not used to replace the prespecified primary analysis. If the direction of the result remained consistent after sensitivity analysis, the result was robust to the specified sensitivity analysis; if the pooled effect size or statistical significance changed after excluding certain studies, differences among studies needed to be fully considered when interpreting that outcome. Subgroup analysis was further conducted according to epilepsy etiology, with emphasis on the efficacy and safety differences between newer-generation and older-generation ASMs in patients with post-stroke epilepsy.

## 3 Results

### 3.1 Literature screening results

According to the prespecified search strategy, a total of 298 records were retrieved, including 286 records from database searches and 12 records supplemented through reference tracing and other sources. After deduplication, 214 records remained and entered title and abstract screening. After final screening, 9 studies were included in the meta-analysis (14–22). The expanded medication search terms did not identify additional eligible comparative studies of lacosamide, eslicarbazepine acetate, or brivaracetam in older-adult populations. The literature screening process is shown in Figure 1.

**Figure 1:**
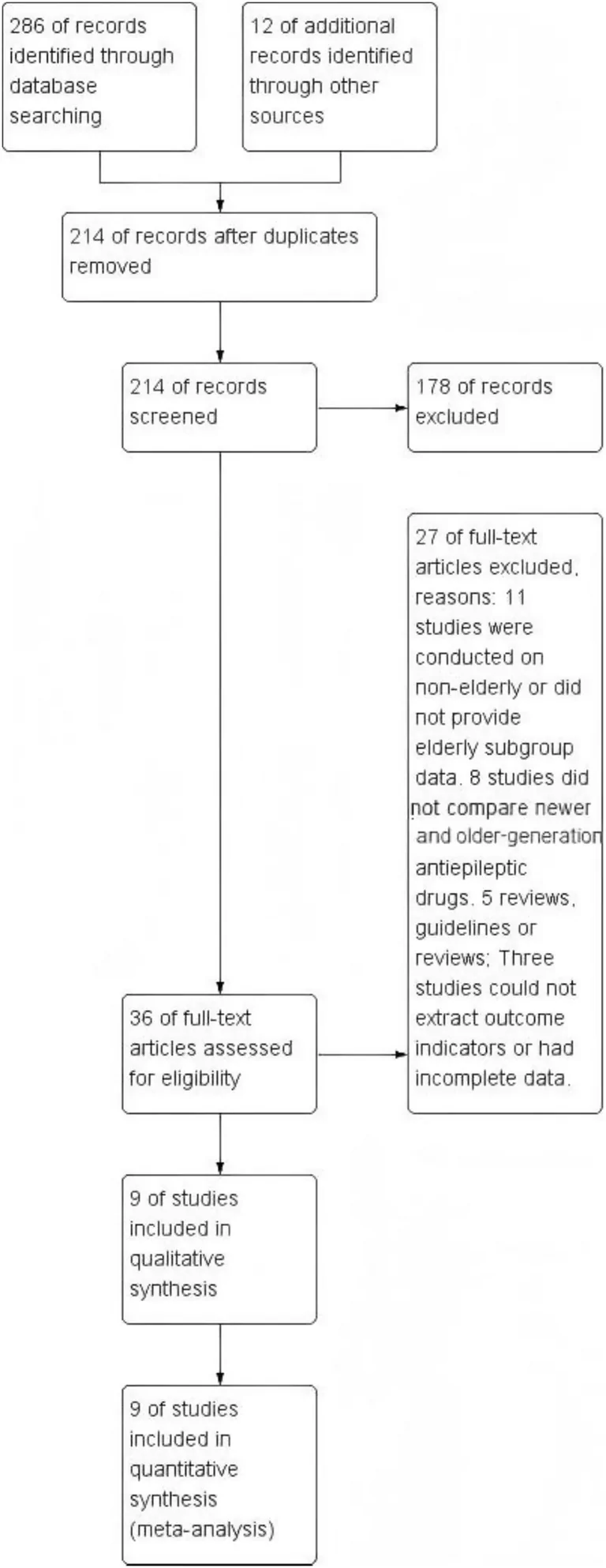
PRISMA 2020 flow diagram of study identification, screening, eligibility assessment, and inclusion.

### 3.2 Baseline characteristics of included studies

The included studies were published between 1999 and 2022, involving a total of 2,507 older patients with epilepsy, including 1,638 patients in the newer-generation group and 869 patients in the older-generation group. Six studies were included in the core main analysis, and two randomized studies were included in the post-stroke subgroup analyses; Arif et al. (22) were retained in the descriptive synthesis but was not pooled because it analyzed multiple medication exposures per patient and did not represent a post-stroke cohort. The study types included randomized controlled trials, open-label randomized studies, *post-hoc* subgroup analyses of randomized studies, and retrospective real-world studies. The main newer-generation ASMs included lamotrigine, levetiracetam, gabapentin, oxcarbazepine, topiramate, and zonisamide; the main older-generation ASMs included carbamazepine, phenytoin, and sodium valproate (Table 1).

**Table 1:** Characteristics, treatment comparisons, and follow-up periods of the included studies.

Author	Age definition	Population	Newer-generation ASM	Older-generation ASM	Follow-up
Brodie et al. (14)	Older cohort; mean age approximately 77 years	Newly diagnosed epilepsy	LTG, *n* = 102	CBZ, *n* = 48	24 weeks
Pohlmann-Eden et al. (15)	≥60 years	Newly diagnosed epilepsy	LEV, *n* = 151	VPA-ER/CBZ-CR, *n* = 152	52 weeks
Qi et al. (16)	≥60 years	New-onset epilepsy	LEV/OXC/LTG, *n* = 233	VPA/CBZ, *n* = 91	1 year
Rowan et al. (17)	Older cohort; mean age approximately 72 years	Newly diagnosed seizures	LTG/GBP, *n* = 395	CBZ, *n* = 198	12 months
Saetre et al. (18)	≥65 years	Newly diagnosed epilepsy	LTG, *n* = 93	CBZ-SR, *n* = 92	40 weeks
Werhahn et al. (19)	≥60 years	New-onset focal epilepsy	LEV/LTG, *n* = 239	CBZ-CR, *n* = 121	58 weeks
Gilad et al. (20)	Older post-stroke cohort	Post-stroke seizures	LTG, *n* = 32	CBZ, *n* = 32	Approximately 12 months
Consoli et al. (21)	Adult post-stroke cohort, predominantly older	Late post-stroke seizures	LEV, *n* = 52	CBZ-CR, *n* = 54	54 weeks
Arif et al. (22)	≥55 years	Older adults with epilepsy	Multiple newer-generation ASMs, *n* = 341	CBZ/PHT/VPA, *n* = 81	12 months

LTG, lamotrigine; CBZ, carbamazepine; LEV, levetiracetam; VPA, valproate/valproic acid; VPA-ER, extended-release valproate; CBZ-CR, controlled-release carbamazepine; OXC, oxcarbazepine; GBP, gabapentin; CBZ-SR, sustained-release carbamazepine; PHT, phenytoin; TPM, topiramate; ZNS, zonisamide; RCT, randomized controlled trial; EpIC, Epilepsia post Ischemic Stroke Study; STEP-ONE, Study in Elderly Patients with Focal Epilepsy.

### 3.3 Quality assessment of included studies

Detailed study-level assessments are presented in Supplementary Table S3. The principal concerns were lack of blinding in open-label trials, *post-hoc* subgroup selection, incomplete control of confounding in retrospective studies, and insufficient reporting of allocation concealment or selective reporting in several older trials. The main outcomes were reported relatively completely, and the grouping information, sample size, and event numbers were sufficient for meta-analysis. Randomized controlled studies were relatively clear in terms of study population source, intervention setting, and outcome reporting, among which double-blind randomized studies had a relatively low risk of bias. Some open-label studies did not implement blinding, which may have increased the risk of performance bias and detection bias. Although Pohlmann-Eden et al. (15) was derived from a randomized study, the data used in the present study came from an older subgroup, which was not the primary target population of the original study; extrapolation of its results should therefore be cautious. Qi et al. (16) and Arif et al. (22) were retrospective cohort or real-world studies, with relatively clear case sources and grouping information. However, because drug allocation was not randomized, treatment selection may have been influenced by baseline disease status, comorbidities, concomitant medication, and physicians’ prescribing preferences, resulting in potential confounding bias. Overall, the included studies did not show obvious missing data for the main outcomes, but open-label design, retrospective data sources, differences in follow-up time, and inconsistent outcome definitions may have affected the stability of some pooled results.

### 3.4 Comparison of seizure freedom rate

Four studies reported seizure freedom rate. The pooled result using a random-effects model showed that seizure freedom occurred in 545/1,018 patients in the newer-generation group and 286/562 patients in the older-generation group. There was no statistically significant difference in seizure freedom rate between the two groups (RR = 1.18, 95% CI: 0.80–1.76, *p* = 0.40). Heterogeneity was substantial (*χ*^2^ = 40.46, df = 3, *p* < 0.00001, *I*^2^ = 93%), indicating that the included studies did not estimate a single clinically uniform effect. The pooled RR should therefore be interpreted as an average across different age definitions, epilepsy populations, medication comparisons, study designs, and outcome windows and not as evidence of equivalence between the two medication categories (Figure 2). Drug-specific, age-threshold-specific, seizure-type-specific, and follow-up-specific meta-analyses were not considered reliable because most potential strata contained only one study or clinically non-comparable comparisons.

**Figure 2:**
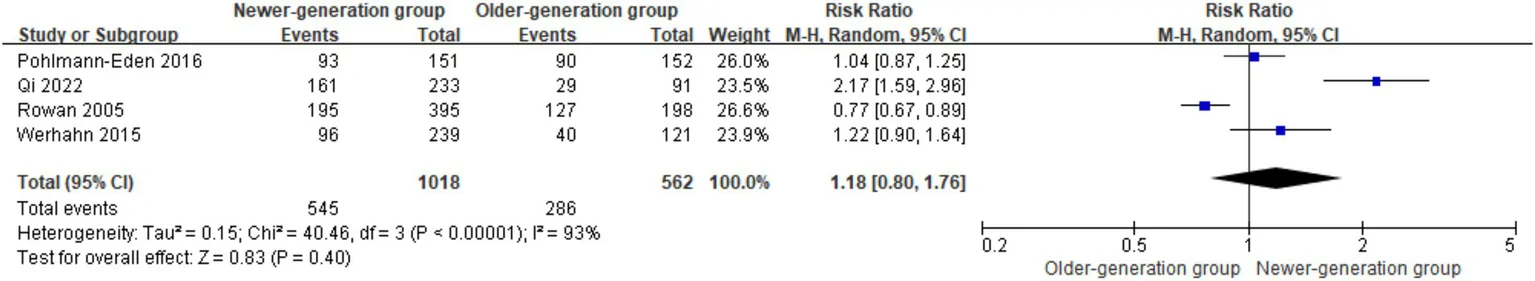
Forest plot comparing seizure freedom rate between the newer-generation and older-generation ASM groups.

### 3.5 Sensitivity analysis of seizure freedom rate

Leave-one-out analyses did not produce a statistically significant pooled effect, although heterogeneity remained substantial after most single-study exclusions. In an additional exploratory restricted analysis, Qi et al. (16) and Rowan et al. (17), which showed markedly divergent estimates in opposite directions, were excluded; the remaining studies yielded RR = 1.11 (95% CI 0.94–1.30, *p* = 0.22, *I*^2^ = 0%). This restricted result should be interpreted as an influence analysis rather than as a replacement for the primary estimate. This result indicated that the high heterogeneity in the original analysis was mainly affected by individual studies. Even after heterogeneity was reduced, however, newer-generation ASMs still did not show a clear advantage in overall seizure freedom rate (Figure 3). After exclusion of the retrospective Qi et al. (16) study, the random-effects estimate for seizure freedom remained non-significant (RR = 0.97, 95% CI 0.74–1.27; *I*^2^ = 82%). Thus, removal of the observational evidence did not resolve the substantial heterogeneity.

**Figure 3:**
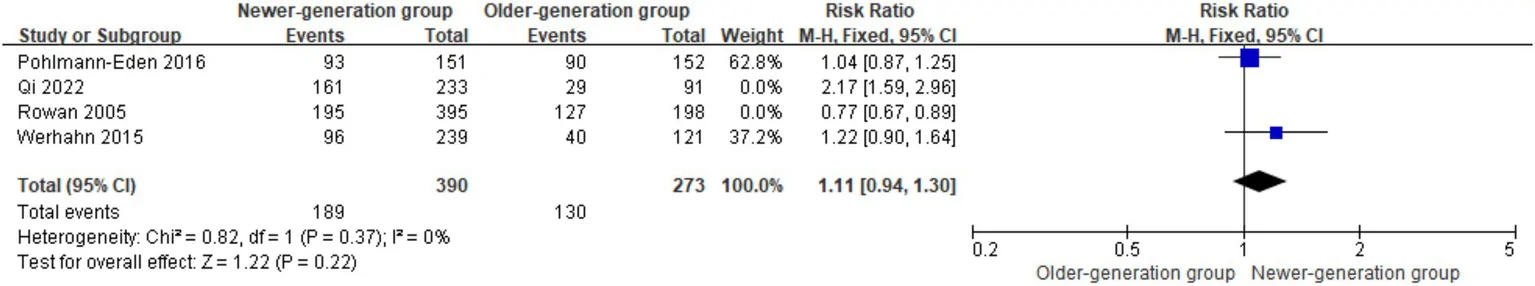
Sensitivity analysis of seizure freedom rate.

### 3.6 Comparison of seizure-free retention rate

Three studies reported seizure-free retention rate. The pooled result using a random-effects model showed no statistically significant difference between the two groups (RR = 1.15, 95% CI: 0.82–1.60, *p* = 0.42). This outcome showed moderate heterogeneity (*χ*^2^ = 5.16, df = 2, *p* = 0.08, *I*^2^ = 61%) (Figure 4).

**Figure 4:**
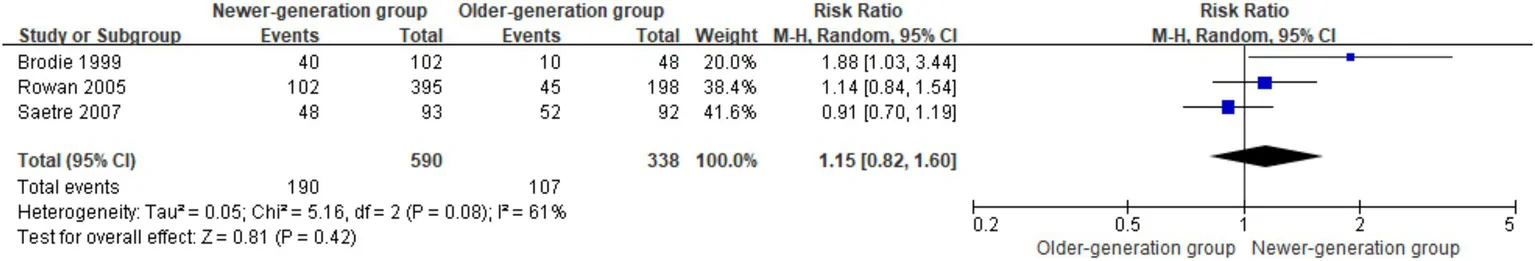
Forest plot comparing seizure-free retention rate between the newer-generation and older-generation ASM groups.

### 3.7 Sensitivity analysis of seizure-free retention rate

Leave-one-out analysis identified Brodie 1999 as the principal influence on heterogeneity. After its exclusion, the pooled estimate remained non-significant (RR = 1.03, 95% CI 0.84–1.27; *p* = 0.76; *I*^2^ = 16%). The complete leave-one-out results are presented in Supplementary Table S5. Therefore, current evidence does not support that newer-generation ASMs are superior to older-generation ASMs in seizure-free retention rate (Figure 5).

**Figure 5:**
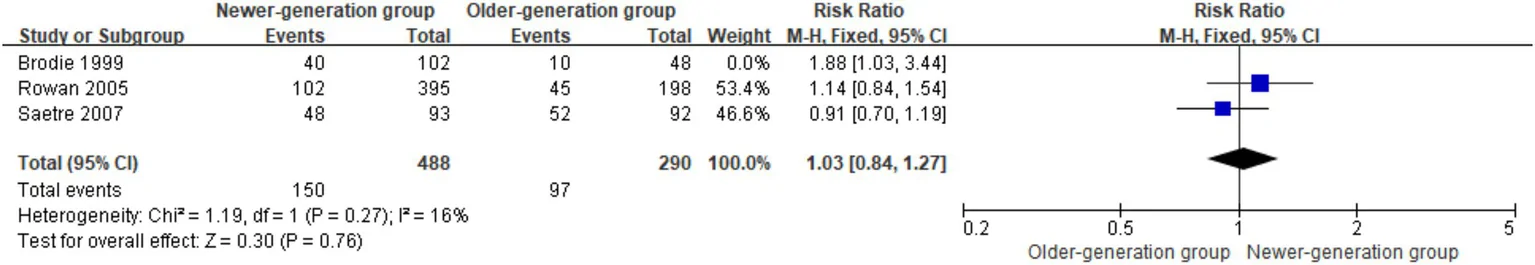
Sensitivity analysis of seizure-free retention rate.

### 3.8 Comparison of treatment retention rate

Five studies were included in the analysis of treatment retention rate. The treatment retention rate in the newer-generation group was higher than that in the older-generation group, and the difference was statistically significant (RR = 1.34, 95% CI: 1.22–1.47, *p* < 0.00001) (Figure 6). A random-effects sensitivity analysis yielded a similar association for treatment retention (RR = 1.30, 95% CI 1.15–1.47), indicating that the direction and statistical interpretation were not dependent on the fixed-effect assumption.

**Figure 6:**
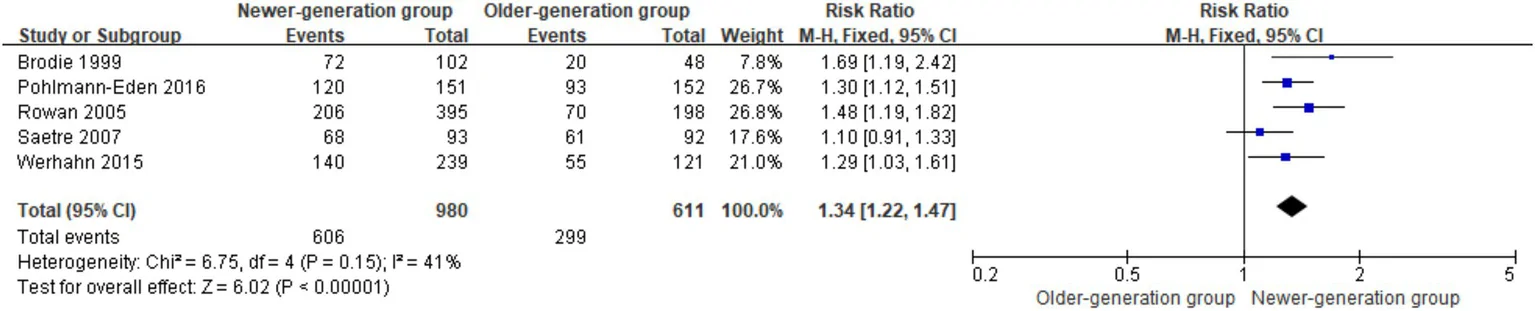
Forest plot comparing treatment retention rate between the newer-generation and older-generation ASM groups.

### 3.9 Comparison of withdrawal rate for any reason

Five studies reported withdrawal rate for any reason. In the newer-generation group, 374/980 patients withdrew, whereas in the older-generation group, 305/611 patients withdrew. The withdrawal rate for any reason in the newer-generation group was lower than that in the older-generation group, and the difference was statistically significant (RR = 0.71, 95% CI: 0.64–0.80, *p* < 0.00001). Because withdrawal for any reason is the inverse of treatment retention, this estimate reflects the same treatment-persistence pattern rather than an independent confirmation (Figure 7). Because *τ*^2^ was estimated as zero, the random-effects sensitivity estimate was identical to the fixed-effect estimate (RR = 0.71, 95% CI 0.64–0.80).

**Figure 7:**
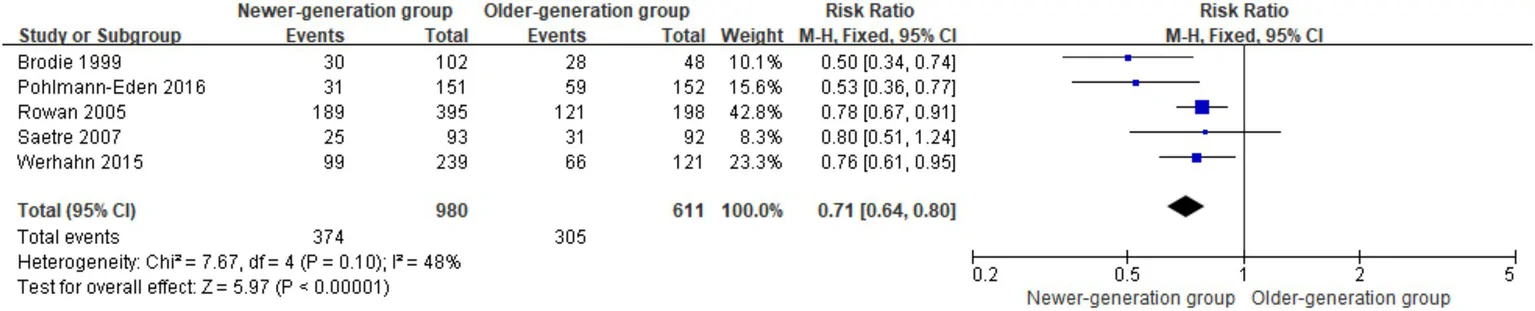
Forest plot comparing withdrawal rate for any reason between the newer-generation and older-generation ASM groups.

### 3.10 Comparison of discontinuation rate due to adverse events

In the newer-generation group, 186/1,213 patients discontinued treatment due to adverse events, compared with 199/702 patients in the older-generation group. The risk of discontinuation due to adverse events was lower in the newer-generation group than in the older-generation group (RR = 0.54, 95% CI: 0.45–0.64, *p* < 0.00001). This finding indicates a lower rate of adverse-event-related discontinuation with newer-generation ASMs in the included studies, rather than establishing a broader class-level advantage in tolerability (Figure 8). Exclusion of Qi et al. (16) from the adverse-event-related discontinuation analysis produced a similar estimate (RR = 0.55, 95% CI 0.46–0.66; *I*^2^ = 0%), indicating that the direction of this association was not dependent on the retrospective study.

**Figure 8:**
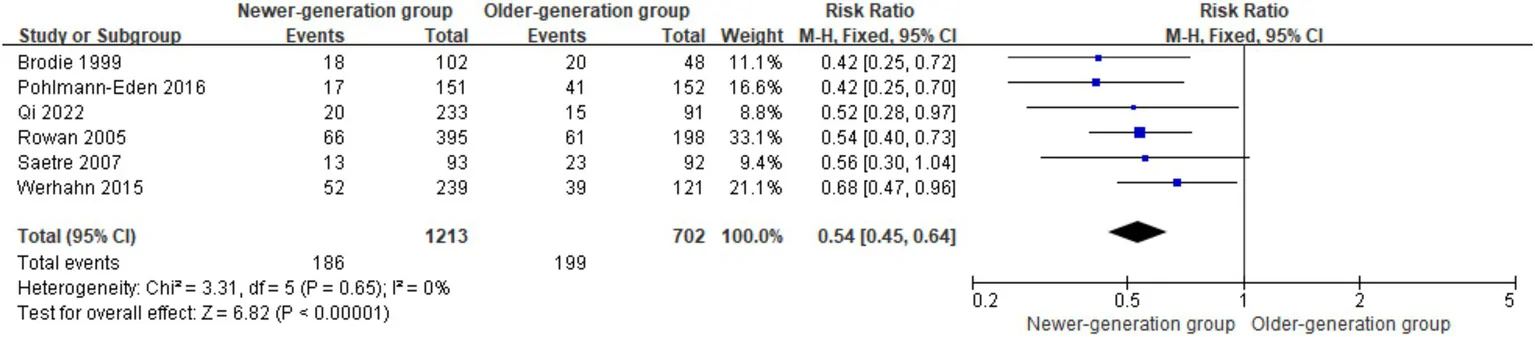
Forest plot comparing discontinuation rate due to adverse events between the newer-generation and older-generation ASM groups.

### 3.11 Publication bias analysis

Fewer than 10 studies contributed to each outcome; therefore, publication bias could not be assessed reliably.

### 3.12 Subgroup analysis

#### 3.12.1 Comparison of seizure freedom rate in the post-stroke epilepsy subgroup

In the post-stroke epilepsy subgroup, two randomized studies reported seizure freedom. Seizure freedom occurred in 72/84 patients in the newer-generation group and 60/86 patients in the older-generation group. Under a random-effects model, the exploratory analysis did not show a statistically significant difference in seizure freedom between newer-generation and older-generation ASMs (RR = 1.30, 95% CI 0.81–2.08; *p* = 0.28). Heterogeneity remained substantial (*χ*^2^ = 4.26, df = 1, *p* = 0.04; *I*^2^ = 77%), precluding a class-level treatment recommendation (Figure 9).

**Figure 9:**
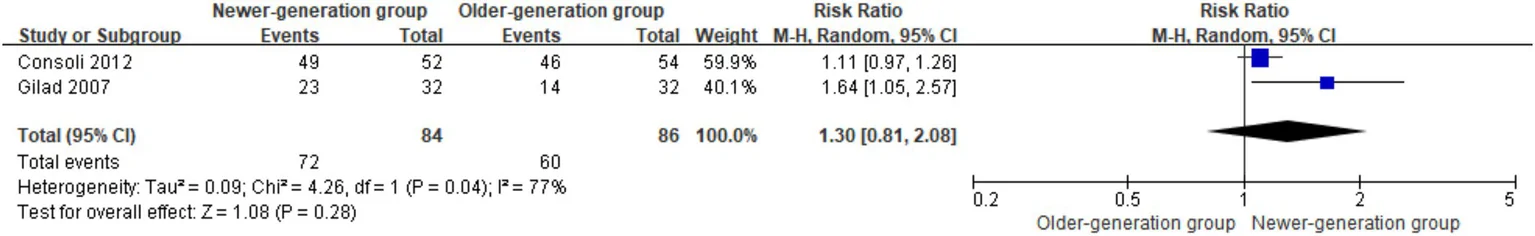
Random-effects meta-analysis of seizure freedom in the post-stroke epilepsy subgroup based on two randomized studies.

#### 3.12.2 Comparison of adverse-event-related discontinuation in the post-stroke epilepsy subgroup

Two randomized studies reported adverse-event-related discontinuation in the post-stroke epilepsy subgroup. Discontinuation due to adverse events occurred in 4/94 patients in the newer-generation group and 14/98 patients in the older-generation group. The random-effects analysis did not show a statistically significant difference between medication categories (RR = 0.31, 95% CI 0.04–2.57; *p* = 0.28), and heterogeneity remained substantial (*χ*^2^ = 2.92, df = 1, *p* = 0.09; *I*^2^ = 66%) (Figure 10).

**Figure 10:**
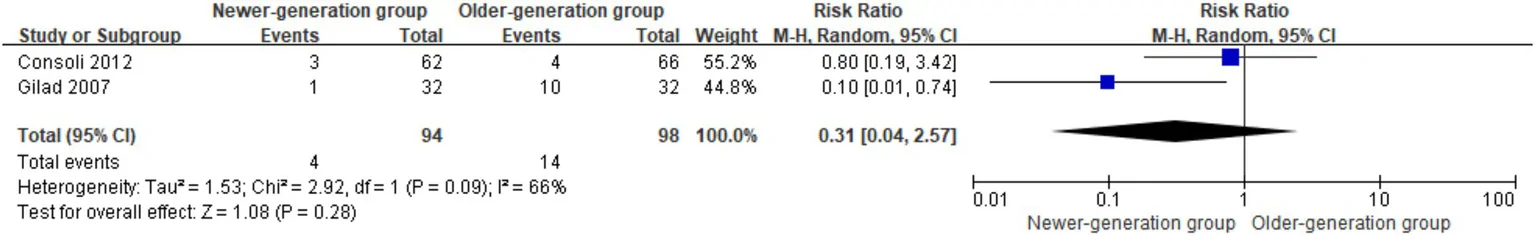
Random-effects meta-analysis of adverse-event-related discontinuation in the post-stroke epilepsy subgroup.

## 4 Discussion

This review found no clear class-level difference in overall seizure freedom or seizure-free retention, whereas treatment retention and adverse-event-related discontinuation favored newer-generation ASMs. These findings arose from nine studies that differed in age definition, epilepsy etiology, medication comparison, study design, follow-up duration, and endpoint definition. Moreover, treatment retention and all-cause withdrawal are inverse outcomes, while adverse-event-related discontinuation is a cause-specific component of withdrawal; these findings should therefore be interpreted as related measures of treatment persistence rather than as independent confirmation of the same association. Several limitations directly constrain interpretation. The newer-generation and older-generation categories contain pharmacologically diverse medications and do not represent internally homogeneous treatment classes. The evidence also combined double-blind trials, open-label trials, *post-hoc* subgroup data, and retrospective cohorts, each with different susceptibility to bias. Age thresholds ranged from 55 to 65 years, while some post-stroke studies enrolled broader adult populations whose participants were predominantly older. The small number of studies prevented reliable stratification by individual medication, age threshold, seizure type, or follow-up duration. In addition, the review was not prospectively registered, publication bias could not be assessed reliably, and certainty of evidence was low or very low. The post-stroke subgroup analyses did not show a significant class-level difference in either seizure freedom or adverse-event-related discontinuation, and substantial heterogeneity limited the interpretation of both outcomes. These findings suggest that the clinical evaluation of drug therapy for epilepsy in older adults should not focus only on whether seizures are controlled, but should also include tolerability, risk of treatment interruption, and feasibility of long-term medication use. The medications included within each operational category were not interchangeable. They differed in mechanism of action, renal or hepatic elimination, enzyme-inducing potential, cognitive and psychiatric effects, and suitability for particular seizure types or comorbidity profiles. Consequently, the pooled estimates may obscure clinically important medication-specific effects and should not be applied uniformly to every ASM within either category.

First, we observed that newer-generation ASMs did not improve overall seizure freedom rate, and this finding is clinically reasonable. Clinically, although older-generation ASMs have problems such as adverse reactions and drug interactions, their antiseizure efficacy has been verified through long-term use. Carbamazepine, sodium valproate, and phenytoin can still provide satisfactory seizure control in focal seizures and in some patients with new-onset epilepsy (23, 24). Therefore, when the outcome is evaluated solely by whether seizure freedom is achieved, newer-generation drugs may not necessarily show a clear advantage. In addition, the included studies differed in epilepsy type, etiological composition, drug regimen, and follow-up duration, and the high heterogeneity in the original analysis also suggested that this indicator was susceptible to differences in study population and outcome definition. The *I*^2^ of 93% indicates that the seizure-freedom studies did not estimate a single clinically uniform effect; accordingly, the non-significant pooled RR should not be interpreted as evidence of equivalence. Although the exploratory restricted analysis also produced a non-significant estimate, it contained only two studies and cannot eliminate the uncertainty arising from the primary heterogeneous evidence base. Previous studies on new-onset epilepsy in older adults have reported similar findings: differences in seizure control among lamotrigine, levetiracetam, carbamazepine, and other drugs were inconsistent across studies, whereas between-group differences were more often reflected in tolerability and discontinuation risk (25). Moreover, in the comparison of seizure-free retention rate, no difference was observed between the two groups, which further suggests that the advantage of newer-generation drugs is not mainly expressed as stronger antiseizure efficacy. Although this indicator is affected by both seizure control and continued medication use, and is theoretically closer to clinical benefit, dose titration speed, discontinuation criteria, changes in comorbidities, and follow-up duration may all influence the result in older patients. Some older-generation drugs can still maintain relatively stable seizure control once an effective dose is reached and the patient can tolerate it (26). Although some newer-generation ASMs may be associated with fewer adverse-effect-related treatment interruptions, their mechanisms of action and suitable patient populations are not entirely the same, and their potential efficacy differences may be diluted in pooled analysis (27). From a pharmacological perspective, the two drug categories act on different targets, including sodium channels, calcium channels, synaptic vesicle proteins, and inhibitory neurotransmitter systems, but both ultimately aim to reduce abnormal neuronal excitability and suppress synchronized discharges (28). We therefore believe that the advantages of newer-generation drugs may lie more in safety, fewer interactions, and better treatment persistence than in a higher absolute seizure freedom rate.

Treatment retention rate was one of the most clinically meaningful findings in this study. It does not merely reflect efficacy; rather, it integrates efficacy, tolerability, adherence, and clinical operability (29). Older patients often have reduced hepatic and renal function, multiple comorbidities, and polypharmacy. The enzyme-inducing properties, narrow therapeutic windows, and high protein binding of older-generation drugs may make blood concentrations more prone to fluctuation and may increase the risks of dizziness, somnolence, ataxia, hyponatremia, and cognitive impairment (30). Even when these adverse reactions are not severe, they may still lead patients to reduce the dose or discontinue treatment, or prompt physicians to switch medication (31). Levetiracetam, lamotrigine, gabapentin, and other newer-generation drugs have relatively stable pharmacokinetic profiles, fewer drug interactions, and more flexible dose adjustment, making them better suited to the practical needs of long-term treatment in older patients (32). We speculate that this is one reason why treatment retention was higher with newer-generation drugs. In other words, the benefit of newer-generation drugs may not come from stronger antiseizure activity, but from their ability to maintain efficacy while reducing factors that interfere with treatment persistence. The all-cause withdrawal and adverse-event-related discontinuation findings should be interpreted alongside treatment retention as related measures, rather than as independent confirmation of the same association. Older patients are more sensitive to central nervous system adverse reactions, and even mild sedation, balance impairment, or cognitive fluctuation may increase the risks of falls, medication errors, and decline in daily functioning (33). Therefore, in the treatment of epilepsy in older adults, safety is not an accessory indicator outside efficacy, but an important prerequisite for whether treatment can be maintained over the long term. Previous real-world studies and related guidelines have also emphasized that drug regimens with fewer interactions, less cognitive impact, and better tolerability should be prioritized in older patients with epilepsy (34).

The post-stroke analysis did not provide reliable evidence of a class-level seizure-control advantage after application of the random-effects model. Although reduced interaction potential may be clinically relevant in patients receiving antiplatelet, anticoagulant, antihypertensive, lipid-lowering, or glucose-lowering therapy, the analysis included only two randomized studies and showed substantial heterogeneity. The result should therefore be regarded as exploratory and hypothesis-generating rather than as support for preferential use of a medication category. However, previous studies have shown that post-stroke patients usually require long-term treatment with antiplatelet drugs, anticoagulant, antihypertensive, lipid-lowering, and glucose-lowering therapies, and older-generation enzyme-inducing ASMs may interfere with the metabolism of these drugs, thereby affecting the stability of secondary prevention for cerebrovascular disease (35). At the same time, post-stroke patients may already have cognitive decline, gait abnormalities, and motor dysfunction. Sedation, dizziness, or ataxia caused by older-generation drugs may further amplify functional impairment (36). Levetiracetam, lamotrigine, and other newer-generation drugs have fewer interactions and relatively milder effects on cognitive and motor function (37), which may be clinically relevant when selecting an individual ASM; however, the present subgroup analysis did not establish a class-level seizure-control advantage. Heterogeneity in this subgroup was high, suggesting that the included studies differed in stroke type, time of seizure onset, diagnostic criteria, follow-up duration, and other aspects. In addition, no significant difference was observed in adverse-event-related discontinuation. Therefore, the subgroup results are more suitable as a reference for drug selection in specific etiological populations, rather than as a fixed priority recommendation for all patients with post-stroke epilepsy.

Because only nine studies were eligible and several outcomes were informed by three to five studies, this review should be viewed as a focused synthesis of older-adult retention and discontinuation outcomes rather than a definitive comparative ranking of individual ASMs. Its findings complement broader comparative reviews by focusing on outcomes particularly relevant to treatment persistence in older populations. Furthermore, the definition of older adulthood varied substantially, the eligible evidence included minimum age thresholds of ≥55, ≥60, and ≥65 years, as well as post-stroke cohorts without a uniform older-adult threshold. These populations may differ in frailty, organ function, comorbidity burden, polypharmacy, seizure etiology, and susceptibility to adverse effects; consequently, part of the between-study variation may reflect population composition rather than medication category. Some studies were open-label studies, *post-hoc* subgroup analyses, or retrospective studies, and selection bias, confounding bias, and detection bias could not be completely avoided. Therefore, although the present study grouped drugs at the strategy level as “newer-generation drugs” and “older-generation drugs” to reflect the overall trend, the mechanisms of action and adverse reaction profiles of different drugs within the same category are not completely consistent. The absence of prospective protocol registration limits the ability to distinguish fully between prespecified and data-informed analytical decisions, particularly for the post-stroke subgroup and influence analyses. More high-quality studies focusing on specific drugs, specific etiologies, and long-term real-world outcomes are still needed.

## 5 Conclusion

Across nine clinically heterogeneous studies, overall seizure freedom and seizure-free retention did not clearly differ between newer-generation and older-generation ASMs, whereas treatment retention and adverse-event-related discontinuation favored newer-generation agents. Treatment retention, all-cause withdrawal, and adverse-event-related discontinuation are related outcomes and should not be interpreted as independent confirmation of a single class-level advantage. These estimates represent average associations across pharmacologically diverse medications and mixed study designs and are limited by risk of bias, variable age definitions, inconsistent outcome windows, and low or very-low certainty of evidence. The post-stroke analyses were exploratory and did not support a class-level treatment preference.

## Data Availability

The original contributions presented in the study are included in the article/Supplementary material, further inquiries can be directed to the corresponding author.
